# The Prophylactic Use of Morphine in Severe Cerebral Injuries: Cerebral-Localisation of the Mental Faculties

**Published:** 1904-07-09

**Authors:** 


### THE PROPHYLACTIC USE OF MORPHINE IN SEVERE CEREBRAL INJURIES: CEREBRAL LOCALISATION OF THE MENTAL FACULTIES.

Mr. J. A. MacDougall[1] relates three cases of cerebral injury—two being attempted suicide by pistol-shot and one a fracture of the base from a fall on the skull—in which the administration of morphine over a period of two to three weeks produced beneficial results. He argues that as the drug quiets the brain cells, lessens pain, and lowers vascular pressure, it will tend in such cases to promote convalescence and to avoid congestive and inflammatory developments. In the first case the bullet had been driven into the skull through the roof of the right auditory meatus. After trephining the skull and the removal of a blood-clot—a quantity of softened brain matter at the same time escaping—the patient's general condition in relation to pulse and respiration rates and mental clearness improved. Morphine was then ordered hypodermically, one-sixth of a grain every six or eight hours, and was continued for at least a fortnight. The patient after the operation had no anxious symptoms and made a complete recovery. In the second case, a pistol-shot in the right frontal region, a considerable portion of the frontal lobe was reduced to pulp, but under similar treatment the patient made an uninterrupted convalescence. The third patient had symptoms of fracture of the base of the skull (middle fossa), and some 48 hours afterwards was very irritable and restless and the subject of delusions. After free purgation, morphine was commenced, and gradually all signs of cerebral irritation subsided, and in the end the patient was able to return to his duties as a coachman. Whilst emphasising the claim for the trephine in suicidal pistol-shot wounds, Mr. MacDougall considers that the morphine treatment had a large share in the satisfactory issue in his cases. He points out how bad is the prognosis in pistol-shot wounds of the brain, and how frequently cerebral laceration is followed by an early and gradually increasing rise of temperature. The absence of this from his cases may, he thinks, be claimed as a result of the administration of morphine. He refers to a paper by Dr. Barr,[2] of Liverpool, on the use of opium in meningitis.

Another point of interest in Mr. MacDougall's article is afforded by the fact that the second patient, a young woman who shot herself in the right frontal region, appears, in spite of the severity of the injury, never to have lost consciousness. According to her statement she expected instantaneous death, but the discharge of the pistol was followed by most intense agony in her head and ear, compelling her to seek her home, fully a quarter of a mile distant, and *en route* she threw the pistol away. Further, it was noted that there were during the progress of the case no mental or emotional disturbance, and the patient's high level of culture and education remained unaffected. Such a record has, in the first place, an obvious medico-legal bearing; it is an illustration of the amount of volitional activity which may be retained in spite of severe injury to the brain. In the second place, the fact that a lesion in the *right* frontal region was unattended by symptoms of mental disturbance has an interesting relationship to the suggestion that it is in the *left* prefrontal lobe that the mental faculties are localised. Phelps[3] has given considerable attention to this point. His personal experience of 800 cases of intracranial traumatism where more than 300 were verified by operation or autopsy offers strong support to the above suggestion. This experience, together with an analysis of other records, shows that with very rare exceptions extensive laceration of the prefrontal region of the left frontal lobe was characterised by abrogation of mental power, whilst more superficial injury manifested itself by mental aberration. On the other hand, there are many cases on record in which severe lesions of the *right* prefrontal lobe have not been attended with any alteration of the mental faculties. Possibly a larger series of observations is needed before presenting an unqualified proposition, but the point is one on which surgeons may be able to lend the physiologists and psychologists considerable aid in their endeavours to determine the site of the cerebral activities which are associated with the manifestation of the mental faculties.

[1] Lancet, June 25, 1904. [2] Brit. Med. Jour., Nov. 18, 1899. [3] Amer. Jour. Med. Sciences, April, 1902 (quoted Brit. Med. Journ., May 17, 1902).

